# Association between condylar asymmetry and temporo- mandibular disorders using 3D-CT

**DOI:** 10.4317/medoral.17786

**Published:** 2012-02-09

**Authors:** Rosa M. Yáñez-Vico, Alejandro Iglesias-Linares, Daniel Torres-Lagares, José L. Gutiérrez-Pérez, Enrique Solano-Reina

**Affiliations:** 1 BDS, MSc (Orthodontics and Dentofacial Orthopaedics). Lecturer Masters Programme in Orthodontics and Dentofacial Orthopaedics. School of Dentistry. University of Seville; 2 DDS, MSc (Orthodontics and Dentofacial Orthopaedics). Lecturer Masters Programme in Orthodontics and Dentofacial Orthopaedics. School of Dentistry. University of Seville; 3 DDS, MSc (Oral Surgery). Professor of Oral Surgery. Department of Stomatology. University of Seville; 4 MD. Professor of Oral Surgery. Chairman of Oral Surgery. Department of Stomatology. University of Seville; 5 MD, MSc (Orthodontics and Dentofacial Orthopaedics). Professor of Orthodontics. Chairman of Orthodontics. Department of Stomatology. University of Seville

## Abstract

Objectives: Using reconstructed three-dimensional computed tomography (3D-CT) models, the purpose of this study was to analyze and compare mandibular condyle morphology in patients with and without temporomandibular disorder (TMD). Study Design: Thirty-two patients were divided into two groups: the first comprised those with TMD (n=18), and the second those who did not have TMD (n=14). A CT of each patient was obtained and reconstructed as a 3D model. The 64 resulting 3D condylar models were evaluated for possible TMD-associated length, width and height asymmetries of the condylar process. Descriptive statistics were used to assess the results and student’s t tests applied to compare the two groups. Results: Statistically significant (p<0.05) vertical, mediolateral and sagittal asymmetries of the condylar process were observed between TMD and non-TMD groups. TMD patients showed less condylar height (p<0.05) in comparison with their asymptomatic counterparts. Conclusions: Using 3D-CT, it was shown that condylar width, height and length asymmetries were a common feature of TMD.

** Key words:**Condilar asymmetry, 3D-computed tomography, X-ray diagnosis , maxillofacial surgery, orthodontics.

## Introduction

Temporomandibular disorders (TMD) constitute one of the most frequent causes of non-dental pain in the orofacial region. The etiology of TMD is multi factorial, being related to factors such as stress (1), muscle hyperactivity (2), arthrogenous factors (3), parafunctions or the anatomy of the temporomandibular joint (TMJ) (4). Many studies have tried to relate TMD to structural factors in the anatomy of this joint (5). Bezuur (6) found that TMJ disorders were influenced by structural factors such as mandibular asymmetry. Habets (5) introduced a method for determining asymmetry between the mandibular condoles, which was based on comparing the vertical heights of the right and left mandibular condyles and the rami. He found that asymmetry of mandibular height correlated significantly for patients with TMJ disorders, compared to those without them. Other authors have corroborated this conclusion, finding asymmetrical height in the condylar process to be a significant relationship in patients with the disorder compared to those who were asymptomatic (7,8). Other researchers, however, were unable to demonstrate this relationship (9), and found no statistically significant differences between condylar asymmetry in TMD patients compared to those with no signs or symptoms of TMD.

Previous studies used conventional radiographic methods. However, it is difficult to examine TMJ using radiographs due to the superimposition of neighboring structures, such as the petrous region of the temporal bone, the mastoid process, and the articular eminence. Computed tomography (CT) imaging has become an alternative to conventional radiographic methods, because it facilitates a high quality image without superimposition, as well as a 3D reconstruction and analysis of the joints for determining the actual dimensions of the structures (10).

The purpose of our study was to study morphology differences of the two condylar processes using 3D-CT imaging and their possible association with TMD. There have been no similar studies carried out with these purposes and characteristics.

## Material and Methods

Subjects

32 subjects, patients at the Virgen del Rocío University Hospital, Seville, Spain were selected for the study. They ranged between 25 and 42 years in age. The present study was carried out with the full knowledge and consent of each subject and in accordance with the ethical principles governing medical research and human subjects, as laid down in the Helsinki Declaration (2002 version, www.wma.net/e/policy/b3.htm). The data has been treated with absolute confidentiality. Methods of gathering and storing data are subject to the Spanish Organic Law governing personal data protection. The Ethical Committee for Experimentation in the University of Seville (Spain) independently approved the procedure. The criteria for inclusion were as follows: that a CT scan had been performed on the subject during diagnostic testing; that the CT was of sufficient quality; and that the patient had given informed consent. The sample was subdivided into two groups based on the presence or absence of pain, joint sounds and a reduced ability to open or close the mouth (11). Subjects with a history of rheumatoid arthritis, osteoarthritis or injuries to the TMJ were excluded. The groups were the control group (n=14) and the TMD group (n=18). 

Processing and image acquisition

A multi-slice helical LightSpeed (General Electric Healthcare, Spain) scanner was used, generating images at 120 kV tube voltage, 120 mA tube current, 8 sec scan time, 0.625 mm slice thickness, to obtain the computed tomography images. Data was stored in DICOM (Digital Imaging and Communications in Medicine) format and transferred to two hard disks with storage capacities of 80 and 150 GB respectively.

To convert DICOM into three-dimensional images, a personal computer (Intel® Core-2 CPU ASUSTeK Computer Inc, Germany) was used, with the Windows XP (Microsoft Corporation, USA) operating system.

Condylar process measurements were performed directly from the converted 3D reconstructions, using VirSSPA software 1.0 (SSPA, Spain). Software development was funded by the Regional Health Ministry for Andalucía and the European Union (FEDER).

Isolating and measuring the condyle

**Figure 1:**
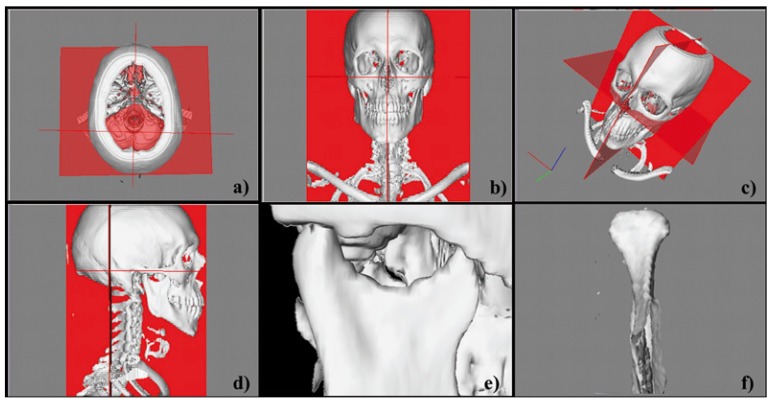
Isolation of 3D-CT reconstruction model. a) Initial axial view 3D reconstruction b) Initial frontal view 3D reconstruction c) Initial free view 3D reconstruction d) Initial lateral view 3D reconstruction e) Horizontal initial sculpting cut f) Complete isolation for condylar measurements

Prior to linear measurements, each of 64 condyles was isolated. A horizontal plane was constructed by creating a plane that linked the bilateral points on the supero-lateral border of the external auditory meatus (SLEAM) and a reference point equidistant to the points located in the centre of each foramen spinosum (ELSA) (12,13) (Fig. 1 1a, 1b, 1c, 1d). An initial cut was made parallel to the horizontal plane until the upper surface of the condyle could be visualized. The remaining surrounding structures were progressively removed using software sculpting tools (Fig.1 1e, 1f).

**Figure 2:**
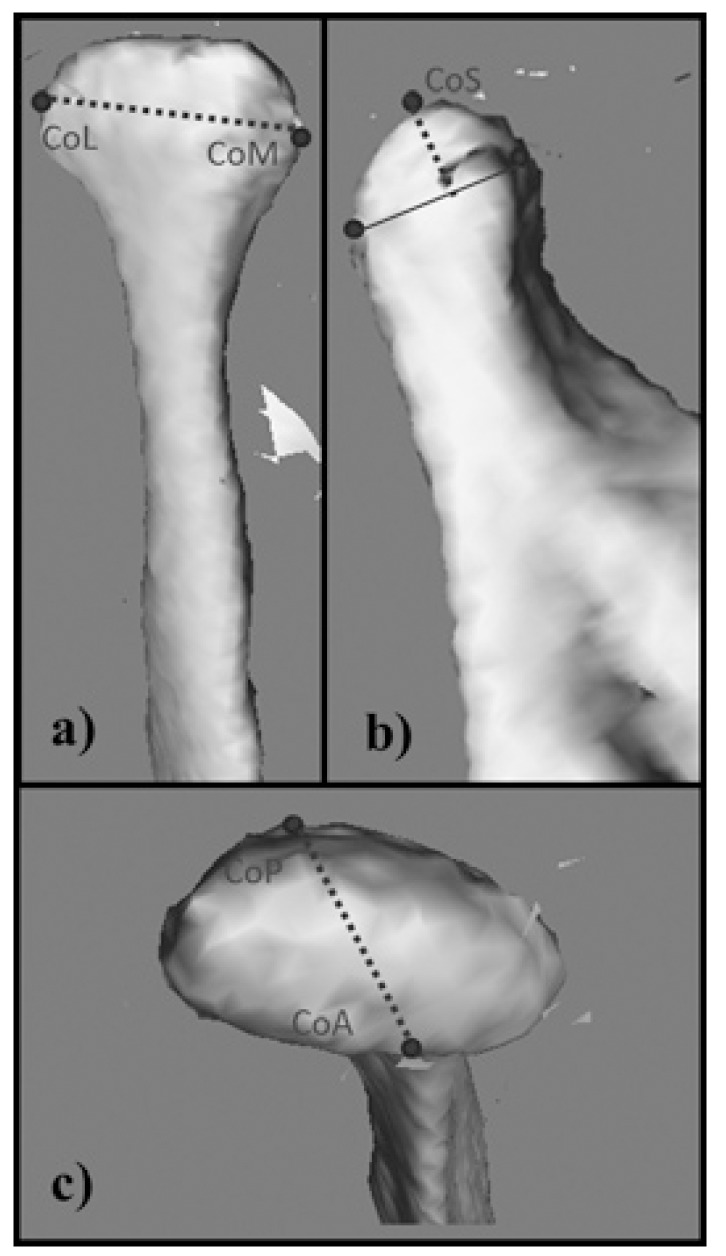
Condyle measurements performed on the 3D-CT model. a)Condylar width (CW) from CoL to CoM b)Condylar height (CH) from CoS to a line linking CoP and CoA, and measured perpendicular to it c)Condylar length (CL) from CoP to CoA

 Table 1 shows the anatomical points for analysis on the 3D models created. Using measurement tools included in the software, the distances (in centimetres) between each of the previously defined anatomical points were measured directly on the 3D model generated. These measurements were defined as follows: condylar width (CW), from CoL to CoM; condylar length (CL), from CoP to CoA; and condylar height (CH), from CoS to a line linking CoP and CoA measured perpendicular to it (Fig. 2). R was measured on the patient’s right side, and L on the patient’s left side. To determine the degree of condylar asymmetry, the following formulae, which were based on previous research (5,7-9), were used:

**Table 1:**
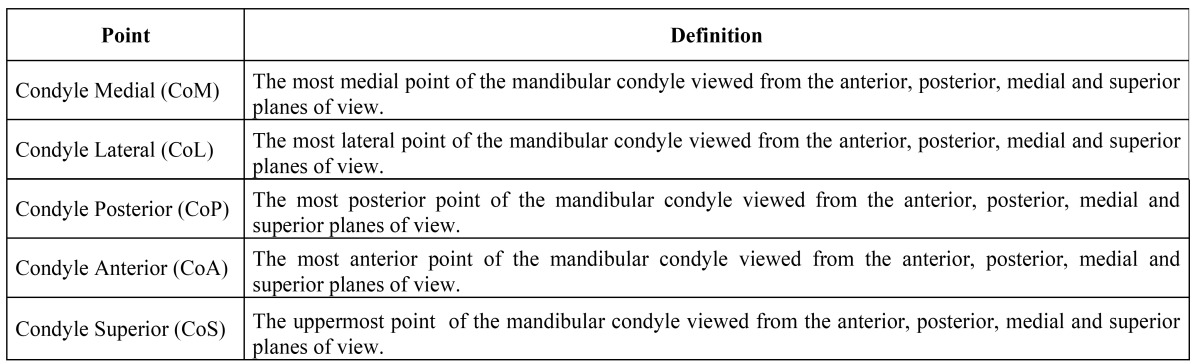
Definitions of anatomical landmarks used to analyze the condylar process.

Width asymmetry = 

Length asymmetry = 

**Figure 3:**
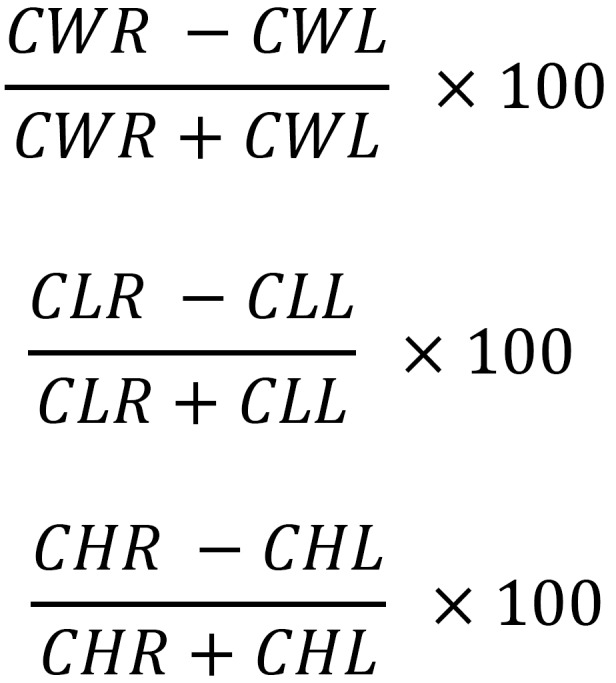
Determine the degree of condylar asymmetry

CWR: right condylar width; CWL: left condylar width; CLR: right condylar length; CLL: left condylar length; CHR: right condylar height; CHL: left condylar height.

Height asymmetry = (Fig. 3)

Reliability of the method

To prevent inter observational error, all the previously defined procedures were carried out by the same operator. Intraobservational error was calculated for a random group of 10 patients drawn from the sample, who were tested twice at an interval of two weeks. The student’s t-test for paired samples was used for calculations, with absence of significance regarded as being indicative of concordance between mean values. An intraclass correlation coefficient of reliability was also calculated. Method error was calculated according to Dahlberg’s formula: SE=√(Σd2/2n), where d is the difference between the double measurements and n the number of paired double measurements (14).

Statistical analysis

The data was analysed using SPSS 17.0 software for Windows (LEAD Technologies, USA).

Univariate analysis of the results consisted of a descriptive analysis of the quantitative variables (mean and standard deviation), and the 95% confidence interval for measurements on the three planes of space. For the bivariate analysis, asymmetry measurement differences between the two groups (control and TMD) were compared, 95% CI, after verifying for randomness and normality (the Shapiro-Wilks test for normality at p>0.05 for all variables in both groups), using the student’s t-test for independent samples (the Wald-Wolfowitz runs test at p>0.05 for all variables in the two groups). Pearson’s correlation coefficient was calculated to evaluate a possible association between age and condylar asymmetry.

## Results

The accuracy of intraobservational error for the condylar width, length and height measurements was 0.04, 0.02, and 0.07 cm, respectively. Likewise, the intraclass correlation coefficient was 0.88, 0.90 and 0.84, respectively, indicating almost perfect agreement (15). Student’s t-test not showed statistically significant differences between original and repeat measurements (p>0.05).

Descriptive statistics for each 3D-CT measurement from the 64 condyles are shown in ( Table 2).

**Table 2:**
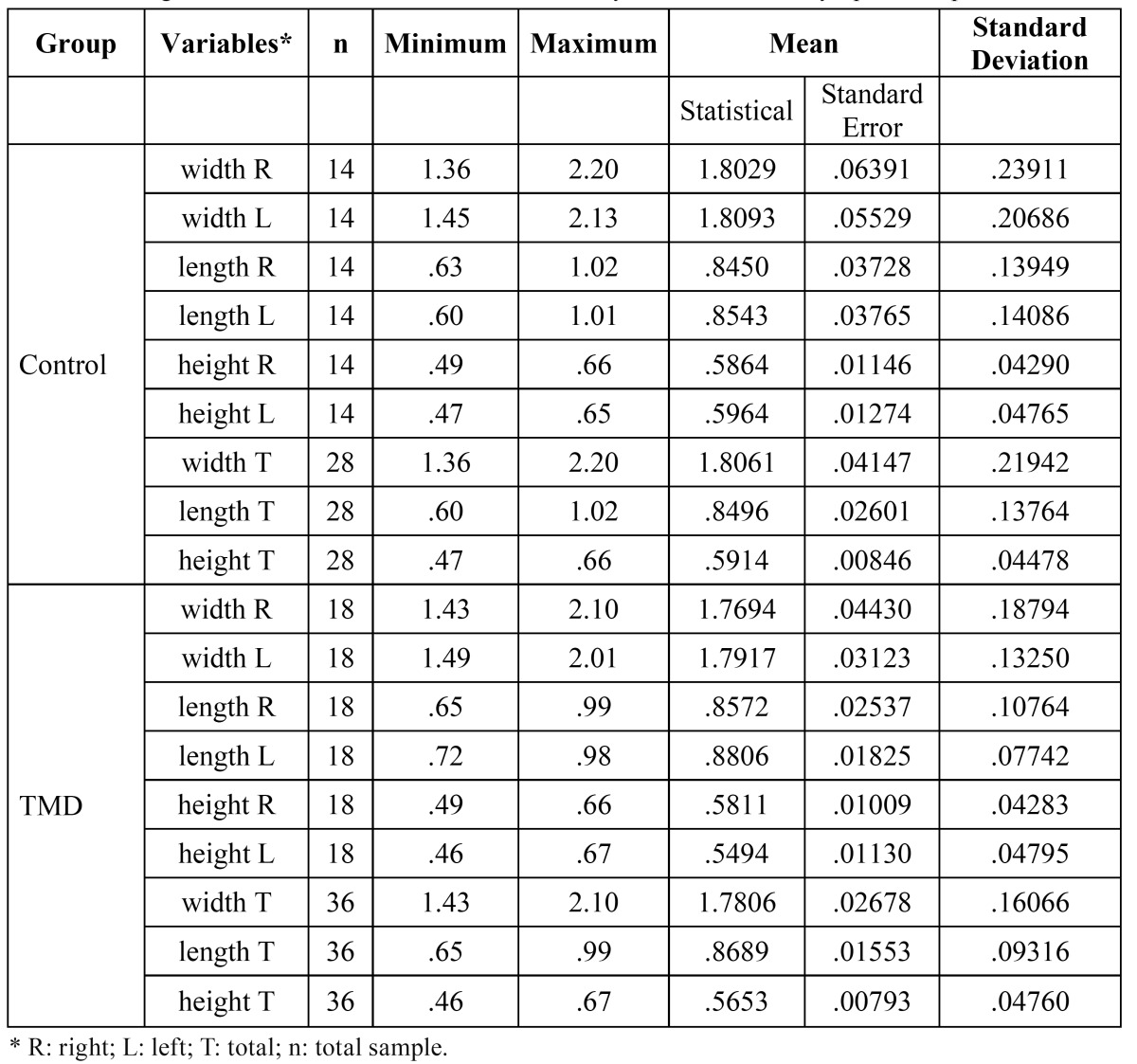
Average measurements obtained for 3D-CT condyles in TMD and asymptomatic patients.

The mean measurement values for total width of the condylar process were 1.81 and 1.78 cm for the control and TMD groups, respectively. The mean total length of the condylar process for the control group was 0.85 cm, and 0.87 cm for the TMD group. The student’s t test for independent samples showed no differences (p > 0.05) of total width and length measurements between the two groups. However, the total height of the condylar process was less in TMD group subjects (p < 0.05) ( Table 3).

**Table 3:**
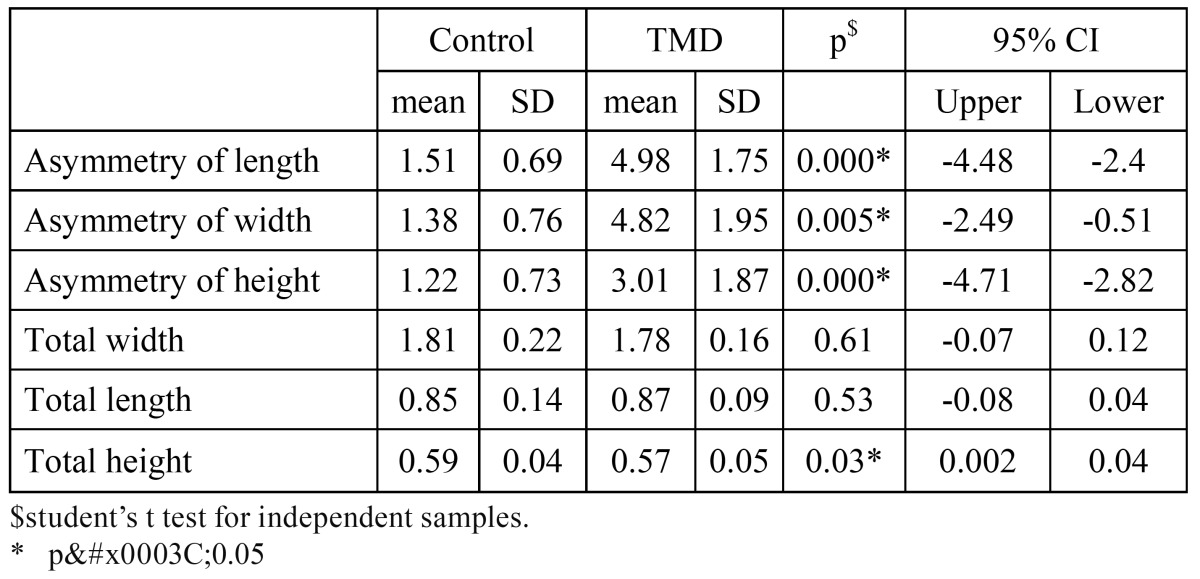
Comparison of condylar asymmetry and 3D measurements in TMD vs. non-TMD groups.

Anteroposterior asymmetry measurements (length asymmetry) of the condylar process showed mean values of 1.51 for the control group and 4.98 for the TMD group (p< 0.05). Mean values for mediolateral asymmetry measurements (width asymmetry) of the condylar processes were 1.38 and 4.82 for the control and TMD groups, respectively. Statistical analysis showed p< 0.05. The mean values for vertical asymmetry, as reflected in the height asymmetry formula, were 1.22 for the control group and 3.01 for the TMD group. The student’s t-test for independent samples showed p < 0.05 ( Table 3). Pearson’s correlation coefficient for age against condylar asymmetry was negative (-0.5) for TMD subjects (p<0.05), but not for control subjects or sagittal and transversal condylar asymmetries (p>0.05).

## Discussion

Overloading the articular surfaces of the temporomandibular joint (TMJ) has been associated with diffe-rences between the right and left mandibular condyles (16). Loads applied to the TMJ could have an influence on its morphology (17,18). Condylar asymmetry leads to greater muscle hyperactivity, which can overload the surface of the joint (3) and, in turn, affect the soft and hard tissue component (13). Shape and function are, therefore, related (18).

Since Habets et al. (5) reported his method for performing symmetry measurements on panoramic x-ray images, several researchers have investigated the relationship between condylar asymmetry and TMD (7-9,19). Miller et al. (19) found a higher mean asymmetry index in the TMD patient group. Other authors also found a relationship between asymmetry and condylar height in panoramic x-rays and TMD patients (7). Saglam and Sanli, however, reported no statistically significant differences in the condylar asymmetry index between patients who had TMD and those who did not (9).

Miller et al. (8) noticed a relationship between age and the condylar asymmetry index of the temporomandibular joint in TMD patients. Other researchers (2,3) confirmed these findings, although Miller and Bodner (20) and Miller and Smidt (21) showed no correlation between condylar asymmetry index and age in a group of patients with Angle’s Class II division 2 and Class III malocclusions.

All the previous studies were carried out using panoramic x-rays. In fact, the panoramic x-ray was conventionally used to compare the vertical condylar height of the two condylar processes. Boratto et al. (22) examined the morphological and radiological data of 100 dry skulls to evaluate the possibility of recognizing mandibular condyle asymmetry using a panoramic radiograph. The results showed no correlation between condylar asymmetry at the anatomical level and radiological asymmetry. Although panoramic x-ray technology has an acceptable cost-benefit ratio due to its minimal radiation exposure, measurements performed with this type of projection alone do not inspire much trust, according to the literature (10). Many inaccuracies are due to distortion and magnification of the ramus and the condyle. Additionally, the condyle structure is frequently superimposed on the lateral edge of the glenoid fossa and the zygomatic arch root.

Nowadays, the use of 3D-CT makes it possible to obtain a more detailed and reliable analysis of condylar morphology by clinical extrapolation. 3D-CT enables us to visualize specific tissues in several sequential planes without the problems of superimposition and magnification. We recently demonstrated, with twenty-one patients, the usefulness of 3D-CT in evaluating mandibular asymmetry (13).

The accuracy of 3D-CT reconstructions has been amply demonstrated. Cavalcanti et al. (23) studied accuracy by comparing the results of linear measurements on 3D-CT images with physical measurements taken on skulls. They concluded that the difference between the two measurements was minimal and that the 3D images were of high precision. In studies using helical CT, Matteson et al. (24) and Hildebolt et al. (25) measured the skull using 3D-CT and reported favourable results. Likewise the accuracy and reliability of mandibular and TMJ dimensions have also been demonstrated (26).

In this study, we assessed intraobservational error, which enables us to confirm the accuracy of the positions of the reference points and the fact that there were no statistical differences between two separate determinations made at suitable intervals of time.

We used 3D-CT throughout to evaluate TMJ. As far as we are aware, there are no previous studies of condylar asymmetry and TMD of a similar nature. Rodrigues et al. (17,18) and Vitral et al. (27,28) investigated the relationship between malocclusion patients and condylar asymmetry on CT axial slice images, although they did not relate this to signs or symptoms of TMD. Recently, 3D-CT was used to assess condylar asymmetry in twenty children with juvenile idiopathic arthritis (JIA). The authors (29) showed that condylar volume and shape could be measured accurately. Condylar asymmetry was found to be a common feature in children with JIA.

Data from the available literature emphasized the relationship between vertical condylar asymmetry and TMD. This agrees with our findings concerning 3D-CT images. In addition we were able to discover a relationship between TMD and width and length differences between the two condyles. It was not possible to demonstrate this previously because the existing literature used conventional two-dimensional radiographic projections, whilst we evaluated the condylar process in three dimensions. In TMD subjects we found statistically significant differences (p<0.05) in mean condylar process height, which was less than in control subjects. This contradicts the suggestion that asymmetry indices would be higher in TMD patients, possibly due to the increase in hard tissue in response to the thickening of the articular surface of the joint as a result of increased loading on this surface (19).

To assess degree of asymmetry, the TMD asymmetry indices relating to height, length and width were compared with those of the control subjects. In our study, statistically significant differences were discovered after the mean asymmetry indices for control and TMD groups had been compared. However, it is still unclear which condylar asymmetry can be considered physiological and what its association with facial asymmetry is.

The results concerning vertical condylar asymmetry in TMD patients coincide with the findings of other authors (3,5,7,8), although the mean vertical asymmetry index for TMD patients reported by the same authors using panoramic x-rays was higher than in our research results ( Table 3). Miller (3) reported a value of 18.76% and the mean age of the group was 25 years; Saglam and Sanli (9) reported a value of 11.11% and the mean age of the TMD group was 26.24 years; Habets (5) obtained a value of 7.3% for patients suffering pain of arthrogenous origin and with a mean age of 35.5 years. There are clearly differences in asymmetry values because of the different methods used. Whereas the panoramic x-ray magnifies structures, the ones we obtained using 3D-CT were real quantitative ones. These differences could also be due to age differences in the subjects under study, with an apparently negative correlation between age and vertical asymmetry. This has in fact been confirmed previously in various studies (2,3,8,9), which corroborate the research shown in this one. Among changes in condylar articular tissue that can be related to age, the superficial and deep zones, the subchondral bone and the intermediate zone all appear to be involved, causing greater depletion of the mesenchymal cells with increasing age. This may be the cause of the osteoarthritic manifestations in TMJ (16,30), although there is no way of knowing ,from the results shown in this study, whether the changes in height were causative or reactive. Future prospective studies are needed in order to assess this.

To sum up, it has been shown that condylar processes can be measured accurately using 3D-CT. Based on the 3D-CT results, condylar morphology—and more specifically, vertical, sagittal and transversal condylar asymmetries—may be associated with TMD. A shorter condylar height was a common feature of TMD patients in comparison with asymptomatic individuals. The present study contributes to enhancing the assessment of condylar processes in relation to TMD.
